# Phage lysin to control the overgrowth of normal flora in processed sputum samples for the rapid and sensitive detection of *Mycobacterium tuberculosis* by luciferase reporter phage assay

**DOI:** 10.1186/1471-2334-13-44

**Published:** 2013-01-28

**Authors:** Balaji Subramanyam, Gomathi Sivaramakrishnan, Azger Dusthackeer, Vanaja Kumar

**Affiliations:** 1 Department of Bacteriology, National Institute for Research in Tuberculosis (Indian Council of Medical Research), Chennai, India

**Keywords:** DLRPA, *M. tuberculosis*, Rapid Diagnosis, Phage lysin

## Abstract

**Background:**

Phage lysin, extracted from three bacteriophages was used in place of antibiotics to control the overgrowth of normal flora in processed sputum samples leading to the sensitive detection of *Mycobacterium tuberculosis* using diagnostic luciferase reporter phage assay (DLRPA).

**Methods:**

A total of 129 sputum samples were processed by modified Petroff’s method. Two Lowenstein Jensen slopes were inoculated from the processed sputum deposit thus obtained. The remaining deposits were transferred to 7 ml of Middlebrook 7H9 complete medium supplemented with phage lysin and incubated at 37°C. DLRPA was done using phAE129 at days 7, 9, 14 and 21. At the end of day 21, the samples were centrifuged and the pellets were inoculated on to 2 more LJ slopes to validate DLRPA results.

**Results:**

The sensitivity and specificity of DLRPA in detecting *M. tuberculosis* from sputum specimens was 90% and 81% respectively compared to conventional LJ culture. The agreement between the methods was 87%. The rate of contamination for DLRPA using phage lysin was 9.3%.

**Conclusion:**

Phage lysin can be used to decontaminate sputum samples for the detection of *M. tuberculosis* by DLRPA directly from processed sputum specimens.

## Background

Lysin, en enzyme produced by bacteriophages, lyses the bacterial cell wall of active host cells to release progeny phages. Though phage lysins are produced inside host bacteria and normally cause lysis from within, they also have external strong lytic activity towards closely related bacteria [1]. Lysins are potential antibacterials regardless of the antibiotic sensitivity of host cells and they exhibit low probability of developing resistance [2]. Phage lysins have been used extensively as an alternative to antibiotics for the targeted killing of bacterial pathogens [3,4].

Phagebiotics consisting of a cocktail of three bacteriophages were used to substitute for antibiotics in liquid media to control the overgrowth of normal flora in processed sputum samples for the rapid recovery of *Mycobacterium tuberculosis*[5]. The phages as such were unable to control some strains of *Bacillus sp* and *Staphylococcus sp*. Moreover, they were temperate in nature leading to the growth of lysogens after 24 hours of phage addition and incubation. To circumvent this situation, phage lysins extracted from all the three phages were pooled and used to supplement phagebiotics for the effective control of normal flora and to prevent the formation of lysogens in processed sputum samples [6]. When phages supplemented with lysins, normal flora were effectively controlled but the lysogens were formed after 24 hours of addition as the lysins were used along with phages. However, the use of phage lysins alone was found to control normal flora as well as to prevent the risk of lysogen development [7].

Pooled phage lysin was evaluated earlier in comparison with antibiotics to control the growth of normal flora in processed sputum samples for the detection of *M. tuberculosis* using BACTEC MGIT 960 system. The results indicated that phage lysin was comparable to antibiotic supplement in decontaminating sputum samples and in detecting *M. tuberculosis*[8]. The present work was aimed at evaluating phage lysin in the rapid detection of *M. tuberculosis* directly from the processed sputum samples using luciferase reporter phage (LRP) assay.

## Methods

### Luciferase reporter phage

Luciferase reporter phage, phAE129 was used for this study to detect *M. tuberculosis* from sputum specimens. The phage was propagated to get a high titer and maintained at 4°C. A titre of 1 x 10^-9^ was used throughout the study.

### Phage lysin

Three bacteriophages namely Chedec 11, Chedec 20 and Chedec 21 were used for the lysin extraction as these phages showed broad host range of infecting more normal flora present in the processed sputum samples [5]. Phage lysin was prepared individually from phages Chedec 11, Chedec 20 and Chedec 21 as described earlier [6]. The clear supernatant fluid with its active lysin obtained during the final stage of lysin preparation was frozen at −20°C [6]. The final precipitate was protein estimated and found to be 0.158, 0.138 and 0. 125 mg/ml respectively for Chedec 11, Chedec 20 and Chedec 21. These phage lysins were pooled prior to use. The working concentration of phage lysin was prepared by mixing 0.8 ml of pooled phage lysins with 7 ml of 7H9 medium supplemented with 0.5% glycerol and 10% albumin dextrose complex (D-glucose-4 grams; NaCl-0.85 gram; Bovine serum albumin-10 grams in 100 ml of distilled water).

### Diagnostic luciferase reporter phage assay (DLRPA) for the detection of *M. Tuberculosis*

Sputum specimens used in this study were obtained from patients enrolled in controlled clinical trials of the National Institute for Research in Tuberculosis (NIRT), Chennai. All the clinical trials conducted at NIRT were approved by institutional ethical committee members and due informed consent was also obtained from patients. A total of 129 sputum specimens were collected; one smear was prepared from each sample and stained by auramine phenol staining method [9]. The specimens were processed by modified Petroff’s method and the deposits thus obtained were washed with 0.067M phosphate buffer saline at pH 7.0 to neutralize the samples. Two Lowenstein Jensen (LJ) slopes were inoculated from the deposits (Petroff’s LJ culture). The LJ slopes were randomized, incubated at 37°C and read every week up to 8 weeks. The remaining deposits were transferred to 7 ml of 7H9 complete medium containing phage lysin and randomized. The samples were incubated at 37°C as a standing culture without shaking. At the end of day 7, 9, 14 and 21, about 500 μl of the samples were withdrawn and mixed with 40 μl of 0.1M CaCl_2_ and 100 μl of phAE129 and incubated at 37°C for 4–6 hours. After incubation, 100 μl of the sample was mixed with 100 μl of 0.33M D-luciferin dissolved in 0.05M sodium citrate buffer at pH 4.5. Relative light unit (RLU) was measured in a luminometer (Monolight 2010) at 10 seconds integration time. RLU was measured in duplicates for all the samples and mean value was used for interpretation of results. A drop of each sample was placed on sheep blood agar plate to check for contamination at every time point. At the end of day 21, all the samples were centrifuged at 3500 RPM for 15 minutes and the pellets were inoculated on to 2 LJ slopes to check whether LRP positives are true positives. The LJ slopes were randomized, incubated at 37°C and read every week up to 8 weeks (Final LJ culture). The results were compared after decoding. The samples resulted as contamination in either of the methods were excluded from the comparison.

### Statistical analysis

The test of significance was calculated by Pearson Chi-Square test using SPSS software version 14.

## Results

Among 129 sputum specimens, 95 samples were positive (74%) and 34 samples were negative (26%) for *M. tuberculosis* by smear using auramine phenol staining method. Likewise, 48 samples were negative (37.2%) and 70 samples were positive (54.3%) by LJ culture and 10 samples resulted in the growth of unclassified mycobacteria (7.8%) while 1 sample was contaminated (0.7%). The sensitivity and specificity of DLRPA for detecting *M. tuberculosis* from sputum specimens was 90% and 81% respectively compared to conventional culture. The agreement between the methods was 87% (Table 1). Either the samples resulted as non tuberculous mycobacteria (NTM) or contamination were excluded from the comparison.

**Table 1:** **Comparison of diagnostic luciferase reporter phage assay (DLRPA) with Petroff’s culture for the detection of *M. tuberculosis***

	**Petroff’s culture**			
		Positive	Negative	Total
	Positive	63	8	71
**DLRPA**	Negative	7	34	41
	Total	70	42	112

Sensitivity 90%; Specificity 81%; Agreement 87%.

Among 48 culture negative samples, 8 samples were positive (16.6%), and 6 samples were contaminated by DLRPA. DLRPA was unable to detect two 1+, two 2+ and three few-colony-growth of culture positive samples (7 out of 70). Among 10 samples that resulted in the growth of NTM, 5 samples were negative and another 5 samples were contaminated by DLRPA. One sample contaminated by conventional LJ culture was also contaminated by DLRPA. The average time to detection for detecting culture negatives, few-colony-growth, 1+ (20–100 colonies), 2+ (>100 colonies) and 3+ (confluent growth) by DLRPA was 19, 19, 15.45, 10.88 and 8.14 days respectively with a median TTD of 14.5 days (Table 2). The earliest time points at which *M. tuberculosis* was detected by DLRPA for each culture grade are given in Table 2.

**Table 2:** **DLRPA for the detection of *M. tuberculosis *from sputum samples**

**Culture grade on LJ**	**No. of samples**	**LRP**			**Early time of detection (days)^@^**				**Average TTD (in days)**	
		**Pos (%)**	**Neg**	**Cont**	**7**	**9**	**14**	**21**	**LRP**	**LJ**
Negatives	48	8 (16.6)	34	6	0	1	1	6	19	NA
Colonies	9	6 (66.6)	3	0	1	0	0	5	19	38.5
1+	13	11 (84.6)	2	0	3	1	1	6	15.45	29.27
2+	26	24 (92.3)	2	0	8	8	5	3	10.88	20.41
3+	22	22 (100)	0	0	17	2	3	0	8.14	14
NTM	10	0	5	5	0	0	0	0	NA	23.1
Cont	1	0	0	1	0	0	0	0	NA	NA

Pos: Positives; Neg: Negatives; Cont: Contaminated; NTM: Non-tuberculous mycobacteria; TTD: Time-to-detection; NA: Not applicable; LJ: Petroff’s culture; Colonies: 1–19 colonies on LJ; 1+: 20–100 colonies; 2+: >100 colonies; 3+: confluent growth of *M. tuberculosis*.

^**@**^ Number of samples detected at early time (i.e. between 7 to 21 days) by D.

DLRPA was found to detect most of the 3+ samples at day 7 while 2+ samples were found to be detected at day 7 and 9. Most of the 1+, few-colony-growth and negatives samples were detected as positive by DLRPA at day 21. Eighty-nine percent (41out of 46) of the higher grade culture positive samples (3+ and 2+) were positive by DLRPA at more than one time points of RLU readings between day 7 and day 21 while 60% (15 out of 25) of the lower grade (1+, few-colony-growth) and culture negative samples were positive only at the end of day 21. The rate of contamination for DLRPA using phage lysin was 9.3%.

The “final LJ culture” confirmed the positives by DLRPA in 64 out of 71 samples. Of the remaining 7 samples, 6 samples were negative and one was contaminated by “final LJ culture”. DLRPA failed to detect 4 samples (two 1+ samples, one 3+ sample and one 1 colony sample) that resulted in growth in “final LJ culture”.

### Statistical analysis

DLRPA was found to be statistically significant compared to conventional culture for the detection of *M. tuberculosis* directly from the processed sputum specimens. The Pearson Chi-Square test showed a value of 0.000. The p value was 0.000001.

## Discussion

Rapid detection, identification and drug susceptibility testing of *M. tuberculosis* is important for better patient management. Delay in diagnosis and treatment is an important contributor for rapid spread of TB in the community [10] leading to excessive morbidity and mortality especially in HIV infected patients [11]. This delay is also responsible for increased nosocomial outbreaks among patients and health care workers [12].

Growing *M. tuberculosis* in liquid medium is much faster when compared to conventional method of detection using solid LJ medium. The major limitation in using liquid medium for the primary isolation of *M. tuberculosis* is the over growth of normal flora which compete for nutrients and mask the growth of tubercle bacilli. Antibiotics are in use either in form of PANTA or PACT to control the growth of normal flora. But it is recommended that exposure of primary isolates to these selective antibiotics should be limited as these agents affect the growth of mycobacteria in liquid media. As an alternative to antibiotics, bacteriophages and their lysins were used in liquid medium to control the growth of normal flora for the detection of *M. tuberculosis*.

Use of liquid culture systems such as BACTEC MGIT 960 has significantly reduced the time for detection and drug susceptibility testing of mycobacteria [13]. The higher initial investment for the equipment and further recurring charges for the procurement of MGIT vials is a major limitation for MGIT 960 system especially in resource limited settings [14]. Phage lysin was evaluated initially in comparison with PANTA to control the overgrowth of normal of normal flora for the recovery of *M. tuberculosis* using BACTEC MGIT 960 system. Phage lysin was found to be comparable to PANTA to control the growth of normal flora.

Mycobacteriophage based methods have been developed for the rapid detection and drug sensitivity testing of *M. tuberculosis*. Among phage based assays, luciferase reporter phage assay has the potential for the detection, identification and drug sensitivity testing of *M. tuberculosis*[15]. LRP assay is utilized to study the drug susceptibility profile of tubercle bacilli using primary culture obtained from LJ medium. The presence of mucus strands, normal flora, enzymes and inhibitory factors in sputum specimens forms the major hurdle in developing a standard diagnostic assay.

Since LRP assay involves frequent opening of the culture vials for taking out aliquots at different days for setting up the assay, it requires stringent measures to prevent the overgrowth of non mycobacterial contaminants especially from environmental organisms. Combination of antibiotics (PANTA) was used in most of the liquid culture detection systems available for mycobacteria to decontaminate processed sputum samples. PANTA was also used to control the overgrowth of normal flora in sputum samples processed for *M. tuberculosis* detection by LRP assay [15,16]. Earlier, lysin was evaluated in comparison with PANTA to control the overgrowth of normal flora normal in processed sputum samples for detection of *M. tuberculosis* by BACTEC MGIT 960 system [8]. Here we reported a simple and bio-friendly methodology to control surviving normal flora using phage lysin in place of antibiotics for luciferase reporter phage assay with improved sensitivity and specificity for the detection of *M. tuberculosis*.

Luciferase reporter phage assay had been recognized for the rapid assessment of drug susceptibility of *Mycobacterium tuberculosis*. The time required for the identification of antibiotics sensitivity pattern of *M. tuberculosis* was reduced from weeks to days using LRP assay [17]. Being a viability based test, LRP was also utilized for the rapid screening of new antituberculosis drugs [18,19]. Mycobacteriophages specific for *M. tuberculosis* alone were used for simultaneous identification and drug susceptibility testing of *M. tuberculosis* cultures grown from MGIT 960 system. Luciferase reporter mycobacteriophage phAE142 was utilized for this purpose [20]. Based on the rapidity, specificity and the ability of LRP assay to detect viable tubercle bacilli, the present work is aimed to improve upon the sensitivity of detection using phage lysin and avoiding the use of antibiotics.

In 2003, Bardarov et al., [16] reported that LRP can be used for the rapid detection of *M. tuberculosis* in conventionally processed sputum samples but the assay required a minimum of >10^7^ CFU/ml as a starting inoculum. Allowing a brief period of time to grow *M. tuberculosis* in liquid media, light can be detected at a rate faster than the doubling time of organisms was also suggested. Accordingly, the samples were incubated at 37°C and tested by LRP infection at each days starting from 1 to 42 to improve the sensitivity of detection. The present approach is also corroborating the earlier methodology but LRP infection by phAE129 is restricted to days 7, 9, 14 and 21 for the detection of *M. tuberculosis*. The results also suggest that the samples containing higher CFU can be detected in ≤ 7 days and the samples with low CFU can be detected between 7 to 21 days. Incubation of samples for more than 21 days probably would result in increased sensitivity of *M. tuberculosis* detection especially from paucibacillary samples.

Phage phAE142 was used for the direct detection of *M. tuberculosis* from processed sputum samples [15]. The results were compared with MGIT 960 and conventional culture on LJ. LRP infection was done on post incubation days 1, 3, 5, 7, 11, 15, 19, 23, 27, 41 and 55. LRP detected *M. tuberculosis* in a range between 1 and 41 days with a mean time to detection of 7 days. The sensitivity of LRP assay was 76% while 97% and 90% of the samples were positive by MGIT 960 and LJ respectively. The sensitivity of detection by LRP was improved from 76% to 92% when contaminated specimens were excluded. In the present work, though the median TTD is 14.5 days, the sensitivity of detecting *M. tuberculosis* is as high as 90% with phAE129 using a simple methodology.

In the present work, 12 out of 129 samples were contaminated by DLRPA when the growth of other mycobacteria is included in the comparison. Among these 12 samples, 5 samples were reported as non-tuberculous mycobacteria (NTM) by conventional LJ method. In principle, all the NTMs also will be considered as contamination by conventional LJ. But because of the limitation of DLRPA to confirm all these NTMs, the samples were reported as contamination. If the NTMs were excluded from the comparison, the rate of contamination for DLPRA would be only 5.4%. Similarly, if consider the total mycobacteria isolated the positivity rate for LJ was 62% (80 out of 129) but for DLRPA it was only 55% (71/129). If the NTMs were excluded, the positivity rate for LJ was 54% and for DLRPA it remains 55%.

Luciferase reporter phage assay has certain advantages over the other liquid based rapid diagnostic assays while detecting *M. tuberculosis* directly from processed sputum samples. The methodology of this rapid assay is very simple and can be easily adopted by any laboratory. The average TTD of “BACTEC MGIT 960”, the current Gold-standard for liquid medium, has been reported to range between 8.5 [21] and 13.3 [22] days. The median TTD of DLRPA is 14.5 as observed in the present study. Among the 10 non-tuberculous mycobacteria grown on LJ, 5 resulted in contamination while growing on liquid medium; the other 5 became negative by DLPRA which reiterates that the phage used in this study is highly specific in infecting *M. tuberculosis*. As only viable bacterial cell will support phage adsorption, infection, replication and subsequent progeny release, the assay detects the active disease. As an indigenous method, propagation, harvest and maintenance of phages are relatively simple and less laborious. The cost-effectiveness of the assay is highly dependent on the cost of luminometer and the substrate, D-luciferin.

## Conclusion

DLRPA, done with phage lysin, is a simple, rapid, highly specific, viability based, bio-friendly, in-house methodology that has the potential to be developed as a point of care system for the rapid detection and drug sensitivity testing of *M. tuberculosis*. DLRPA can be implemented for the rapid availability of diagnostic results for *M. tuberculosis* especially in countries endemic to tuberculosis and in resource limited settings.

## Abbreviations

LRP: Luciferase Reporter phage; DLRPA: Diagnostic Luciferase Reporter Phage Assay; TTD: Time To Detection; CFU: Colony Forming Unit; PANTA: Polymixin B, Amphotericin B, Nalidixic acid, Trimethoprim, Azlocillin; PACT: Polymixin B, Amphotericin B, Carbenicillin, Trimethoprim; MGIT: Mycobacterial Growth Indicator Tube.

## Competing interests

The authors declare that they have no competing interest.

## Authors’ contributions

BS was responsible for the laboratory work and writing of the manuscript. GS was for responsible for the interpretation of the results and data analysis. AD was responsible for the data interpretation. VK was responsible for the study design and overall supervision. All authors read and approved the final manuscript.

## Pre-publication history

The pre-publication history for this paper can be accessed here:

http://www.biomedcentral.com/1471-2334/13/44/prepub
